# Characterization of Mechanisms Lowering Susceptibility to Flumequine among Bacteria Isolated from Chilean Salmonid Farms

**DOI:** 10.3390/microorganisms7120698

**Published:** 2019-12-14

**Authors:** Christopher Concha, Claudio D. Miranda, Luz Hurtado, Jaime Romero

**Affiliations:** 1 Laboratorio de Tecnología Enzimática para Bioprocesos (TEB), Universidad de La Serena, La Serena 1700000, Chile; christopherconchapujado@gmail.com; 2 Laboratorio de Silvigenómica y Biotecnología (SILGENBIO), Universidad de La Serena, La Serena 1700000, Chile; 3 Laboratorio de Patobiología Acuática, Departamento de Acuicultura, Universidad Católica del Norte, Coquimbo 1780000, Chile; luzhurtado21@hotmail.com; 4 Centro AquaPacífico, Coquimbo 1780000, Chile; jaime.m.romero@gmail.com; 5 Laboratorio de Biotecnología, Instituto de Nutrición y Tecnología de los Alimentos (INTA), Universidad de Chile, Macul, Santiago 7810000, Chile

**Keywords:** quinolone resistance, GyrA, mutations, efflux pumps, salmon farm, Chile

## Abstract

Despite their great importance for human therapy, quinolones are still used in Chilean salmon farming, with flumequine and oxolinic acid currently approved for use in this industry. The aim of this study was to improve our knowledge of the mechanisms conferring low susceptibility or resistance to quinolones among bacteria recovered from Chilean salmon farms. Sixty-five isolates exhibiting resistance, reduced susceptibility, or susceptibility to flumequine recovered from salmon farms were identified by their 16S rRNA genes, detecting a high predominance of species belonging to the *Pseudomonas* genus (52%). The minimum inhibitory concentrations (MIC) of flumequine in the absence and presence of the efflux pump inhibitor (EPI) Phe-Arg-β-naphthylamide and resistance patterns of isolates were determined by a microdilution broth and disk diffusion assays, respectively, observing MIC values ranging from 0.25 to >64 µg/mL and a high level of multi-resistance (96%), mostly showing resistance to florfenicol and oxytetracycline. Furthermore, mechanisms conferring low susceptibility to quinolones mediated by efflux pump activity, quinolone target mutations, or horizontally acquired resistance genes (*qepA*, *oqxA*, *aac(6′)-lb-cr*, *qnr*) were investigated. Among isolates exhibiting resistance to flumequine (≥16 µg/mL), the occurrence of chromosomal mutations in target protein GyrA appears to be unusual (three out of 15), contrasting with the high incidence of mutations in GyrB (14 out of 17). Bacterial isolates showing resistance or reduced susceptibility to quinolones mediated by efflux pumps appear to be highly prevalent (49 isolates, 75%), thus suggesting a major role of intrinsic resistance mediated by active efflux.

## 1. Introduction

The Chilean salmon farming industry is commonly affected by an important number of bacterial diseases causing high mortalities, consequently prompting the necessity of using high amounts of antibiotics to ensure salmon production [1,2]. However, it is well known that intensive use of antibiotics is responsible for therapy failures, probably due to a reduction in the susceptibility to antibacterials of the bacterial pathogens associated with this industry [2,3,4,5,6,7].

Quinolones are one of the most important classes of antimicrobials used in human therapy, but their use has been compromised by the increasing emergence of resistant isolates, becoming a prevalent clinical problem [8,9,10]. Quinolones inhibit the activity of DNA gyrase and topoisomerase IV, two essential bacterial enzymes that modulate the chromosomal supercoiling required for critical nucleic acid processes [11,12]. The main resistance mechanisms that additively contribute to quinolone resistance include one or a combination of target-site gene mutations that alter the drug-binding affinity of target enzymes, mutations that lead to reduced intracellular drug concentrations by either decreased uptake or increased efflux, and plasmid-encoded resistance genes that produce target protection proteins, drug-modifying enzymes, or multidrug efflux pumps [13,14,15,16].

Although quinolones are intensively used in human therapy, they are still currently used in aquaculture [2,17,18], and although the worldwide use of quinolones in the aquaculture industry has drastically decreased, the occurrence of bacteria resistant to this antibiotic group in fish farms has been previously detected [19,20,21]. Furthermore, quinolone resistance associated with target protective enzymes mainly encoded by the *qnr* genes as well as the quinolone-modifying enzymes encoded by the *aac(6′)-lb-cr* gene and the quinolone efflux pumps encoded by the *qepA* and *oqxAB* genes, which are usually associated with plasmids, have been previously detected among bacteria isolated from fish farm-associated environments [22,23,24,25]. To a greater extent, quinolone resistance has been described in several pathogenic bacterial species [22,26,27,28], but it has never been found in fish pathogenic species isolated from diseased fish.

Currently, the intracellular bacteria *Piscirickettsia salmonis* causes the highest rates of mortalities in Chilean marine farms [29], and the current absence of effective vaccines to prevent the high mortalities caused by this pathogen in the Chilean salmon farming industry has prompted the necessity of using large quantities of antimicrobials [1,2]. It should be noted that between 2005 and 2010, quinolones were widely used in Chilean salmon farms, reaching approximately 560 tons [2,30], but their use has been reduced considerably, with flumequine being the most used quinolone in Chilean salmon farms [1].

The emergence and dissemination of antibiotic-resistant bacteria in the fish farm-associated aquatic environments can be a serious threat for this industry. Thus, studies to advance a comprehensive knowledge of the mechanisms involved in quinolone resistance in the microbiota associated with fish culture will be of great value to develop efficient strategies to reduce antimicrobial resistance in Chilean salmon farms to prevent future therapy failures as well as to reduce the probability of their spread to the humans.

## 2. Materials and Methods

### 2.1. Bacterial Isolates and Culture Conditions

A total of 65 isolates exhibiting resistance, reduced susceptibility, or susceptibility to flumequine were included in the study. The bacterial isolates used in this study were recovered from various sources of land-based and lake-based Chilean salmonid farms, as previously described [31]. Isolates from land-based culture centers were isolated from various sources including unmedicated fish food pellets, mucus of healthy salmonid fingerlings, and water samples from fish farm influents, effluents, and fish rearing tanks. Isolates recovered from lake-based salmonid cultures were isolated from samples of mucus and intestinal content of healthy reared fingerlings, surface water samples from salmon cages, and samples of sediments beneath salmonid cages. Isolates were obtained from a collection of bacteria obtained from various salmonid farms. Isolates were previously recovered using plates with Tryptic soy agar (Difco labs) containing oxytetracycline (30 μg/mL) or florfenicol (30 μg/mL) and incubated at 22 °C for 5 days. Isolates were stored at −85 °C in CryoBank^TM^ vials (Mast Diagnostica, Reinfeld, Germany) and were grown in Trypticase soy agar (Oxoid, Hants, UK) at 22 °C for 24 h prior to use.

### 2.2. Identification of Isolates

Isolates were cultured in Tryptic soy broth (Oxoid, Hants, UK) at 22 °C for 12–24 h and centrifuged at 9000 g for 3 min using an Eppendorf 5415D microcentrifuge to obtain a pellet. DNA extraction was carried out using the Wizard Genomic DNA Purification commercial kit (Promega, Madison, WI, USA) following the supplier’s instructions, and the obtained DNA samples were stored at −20 °C until analysis. The amplification of the 16S ribosomal genes of the isolates was carried out by PCR, following the methodology described by Opazo et al. [32]. The resulting amplified PCR products were sequenced by Macrogen (Rockville, MD, USA) using the ABI PRISM 373 DNA Sequencer (Applied Biosystems, Foster City, CA, USA). The sequences were edited and matched to the Ribosomal Database Project [33] to identify the bacterial isolates. Isolates exhibiting in their 16S rRNA gene sequence a similarity score of ≤99.4% with a nearest neighbor were not identified to the species level.

### 2.3. Minimum Inhibitory Concentrations (MICs) of Flumequine

The minimum inhibitory concentrations (MICs) of isolates to flumequine were determined by a broth microdilution method according to the Clinical and Laboratory Standards Institute (CLSI) guideline M07-A10 [34]. Conical bottom 96-well microplates containing 0.1 mL of Mueller-Hinton broth (BBL-Becton Dickinson, Cockeysville, USA) were inoculated in triplicate with duplicate concentrations of flumequine (Sigma-Aldrich, Darmstadt, Germany) ranging from 0.0625 µg/mL to 64 µg/mL. Bacterial culture suspensions grown at exponential phase were adjusted at a 0.5 McFarland turbidity (1 × 10^8^ CFU/mL), and an aliquot of 0.01 mL of each bacterial suspension was inoculated into each well in triplicate. Microplates were incubated at 22 °C for 24 h according to CLSI guidelines [35]. The turbidity of the medium in each well was measured by using the Mindray MR-96A microplate reader at an optical density of 600 nm. The MIC was defined as the lowest concentration of flumequine capable of inhibiting visible growth in at least two wells. Three wells without the antibiotic were used as controls for bacterial growth for each strain, and *Escherichia coli* ATCC 25922 was used as a control strain as recommended by the CLSI [34]. Considering that no MIC breakpoints for flumequine are currently stated, we categorized the isolates using as a reference the flumequine epidemiological cut-off (ECOFF) value stated by European Committee on Antimicrobial Susceptibility Testing (EUCAST) [36] for *Escherichia coli* and *Salmonella* spp. (≤2 µg/mL for susceptible). We decided to consider susceptibility, reduced susceptibility, and resistance as those isolates exhibiting MIC values of ≤2 µg/mL, 4–8 µg/mL, and ≥16 µg/mL, respectively.

### 2.4. Antibacterial Resistance Patterns

The susceptibility of isolates to various antimicrobials was determined by a disk diffusion test according to the CLSI [37]. Briefly, the bacterial isolates were resuspended in phosphate buffered saline (PBS) to obtain a turbidity corresponding to 0.5 McFarland standard (bioMerieux, Marcy-l’Etoile, France). Bacterial suspensions were seeded in plates containing Mueller-Hinton agar (MH, Difco Labs, NJ, USA) and the suspension excess was discarded using a micropipette, and disks (Oxoid, Basingstoke, Hampshire, England) containing the following antibiotics were used: cefotaxime (CTX, 30 μg), streptomycin (S, 10 μg), gentamicin (CN, 10 μg), kanamycin (K, 30 μg), oxytetracycline (OT, 30 μg), chloramphenicol (CM, 30 μg), florfenicol (FFC, 30 μg), oxolinic acid (OA, 2 μg), flumequine (UB, 30 μg), enrofloxacin (ENR, 5 μg), furazolidone (FR, 100 μg), and sulfamethoxazole trimethoprim (SXT, 25 μg). Plates were incubated at 22 °C for 24 h according to CLSI guidelines [38], and isolates were considered resistant according to the criteria established by the CLSI [34,39] or by Miranda and Rojas [20]. *Escherichia coli* ATCC 25922 was used as a quality control strain, as recommended by the CLSI [37]. A number of isolates (30%) were re-examined to check the reproducibility of the assay.

### 2.5. Detection of the Activity of Efflux Pumps

To detect the presence of efflux pump activity on flumequine, a modified methodology for the broth microdilution method described by Fernández-Alarcón et al. [40] was used. Briefly, the MIC assay was determined by a microdilution method according to the Clinical and Laboratory Standards Institute (CLSI) guideline M07-A10 [34] and as previously described in the minimum inhibitory concentrations (MICs) in the flumequine section, but MIC assays were performed in the absence and the presence of 20 µg/mL of the broad spectrum efflux pump inhibitor (EPI) Phe-Arg β-naphthylamide (CAS 100929-99-5, Santa Cruz Biotechnology Inc., USA). *E*_max_ values were calculated, corresponding to the ratio between MIC without EPI and MIC in the presence of EPI [41]. *Escherichia coli* ATCC 25922 was included as a control strain, as recommended by the CLSI [34].

### 2.6. Detection of Mutations in DNA Gyrase and Topoisomerase IV Genes

Only isolates categorized as resistant and exhibiting a high MIC value (MIC ≥16 µg/mL) were considered for detection of mutations in the quinolone targets. To detect the presence of chromosomal mutations among these isolates, the sequence of the quinolone resistance determining region (QRDR) of the enzyme DNA gyrase (*gyrA* and *gyrB* genes) and the QRDR homologous section of the topoisomerase IV enzyme (*parC* and *parE* genes) were amplified by using the primers described in Table 1. The amplification conditions were as follows: for *gyrA*, denaturation at 94 °C for 5 min; 35 cycles of denaturation at 94 °C for 1 min, annealing at 55 °C for 1 min, and elongation at 72 °C for 1 min; and a final extension at 72 °C for 7 min; and for *gyrB*, denaturation at 95 °C for 5 min; 30 cycles of denaturation at 95 °C for 30 s, annealing at 49 °C for 30 s, and elongation at 72 °C for 1 min; and a final extension at 72 °C for 7 min. The amplification conditions used for *parC* and *parE* were denaturation at 95 °C for 5 min; 30 cycles of denaturation at 95 °C for 30 s, annealing at 54 °C for 30 s and elongation at 72 °C for 30 s; and finally extension at 72 °C for 7 min. The amplified PCR products were sequenced by Macrogen (Rockville, MD, USA); the amino acid sequences were obtained by using the BioEdit version 7.2.5 software (Ibis Therapeutics, Carlsbad, CA, United States) [42] and compared with the sequences described for the control strain *Escherichia coli* ATCC 9637 (GenBank: CP002185). For GyrA and ParC, a comparison from codon 69 to 110 was performed, whereas for GyrB and ParE, a comparison from codon 394 to 430 was performed.

### 2.7. Detection of Genes Encoding for Quinolone Resistance

All isolates were assayed for the presence of genes encoding for quinolone resistance. The presence of the *qepA* and *oqxA* genes, encoding for efflux pumps; the *aac(6′)-Ib-cr* gene, encoding for an aminoglycoside acetyltransferase that confers resistance by inactivating the antibiotic; and the *qnr* (*qnrA*, *qnrB*, *qnrC*, *qnrD* and *qnrS*) genes, encoding for Qnr proteins, conferring DNA gyrase protection were detected by using the methodology described by Albert et al. [54] using the primers shown in Table 1. The amplification conditions were as follows: for the *qepA* gene, denaturation at 95 °C for 5 min; 45 cycles of denaturation at 95 °C for 30 s, annealing at 54 °C for 30 s, and elongation at 72 °C for 30 s; and finally extension at 72 °C for 7 min. For the *oqxA* gene, denaturation at 95 °C for 5 min; 45 cycles of denaturation at 95 °C for 30 s, annealing at 57 °C for 30 s, and elongation at 72 °C for 30 s; and finally extension 72 °C for 7 min. For the *aac(6′)-Ib-cr* gene, denaturation at 95 °C for 5 min; 35 cycles of denaturation at 95 °C for 30 s, annealing at 57 °C for 30 s, and elongation at 72 °C for 30 s; and finally extension at 72 °C for 30 min. For the *qnrA*, *qnrB* and *qnrS* genes, denaturation at 95 °C (5 min), 35 cycles of 95 °C (30 s), 51 °C (30 s), and 72 °C (30 s); and finally extension at 72 °C (7 min). For the *qnrC* gene, denaturation at 95 °C (5 min), 35 cycles of 95 °C (30 s), 48 °C (30 s), and 72 °C (30 s); and finally extension at 72 °C (7 min). For the *qnrD* gene, denaturation step at 95 °C (5 min); 35 cycles of 95 °C (30 s), 50 °C (30 s), and 72 °C (30 s); and finally extension at 72 °C (7 min). The amplified PCR products were sequenced by Macrogen (Rockville, MD, USA), and genes were identified by a computational analysis of BLAST sequence alignment against the gene sequences included in the GenBank database.

### 2.8. Statistical Analysis

Significant differences between the presence of efflux systems and chromosomal mutations in the DNA gyrase of the isolates were determined by a proportion analysis [55] using the free access software RStudio version 1.2.5001 (RStudio Inc.). Analyses were carried out for all isolates included in the study as well as only for the flumequine-resistant isolates. Differences with a *p* ≤ 0.05 were considered significant.

## 3. Results

### 3.1. Bacterial Identification

Among the 65 isolates included in the study, a predominance of isolates belonging to various species of the genus *Pseudomonas* (34 isolates, 52%) and, to a lesser extent, some enteric species belonging to the *Kluyvera* (six isolates, 9%), *Citrobacter* (five isolates, 7%), and *Hafnia* (three isolates, 4%) genera were detected, as shown in Table 2. Sequences of amplified 16S rRNA genes of isolates were included in the GenBank database, and their accession numbers are included in Table 2.

It must be noted that among the isolates exhibiting MIC values of flumequine of ≥16 µg/mL, a high predominance (88.2%) of representatives of the genus *Pseudomonas* were observed, with the exception of the enteric species identified as *Rahnella aquatilis* and *Kluyvera intermedia*, which exhibited the highest flumequine MIC values (Table 2).

### 3.2. Antimicrobial Resistance of Isolates

**Figure 1:**
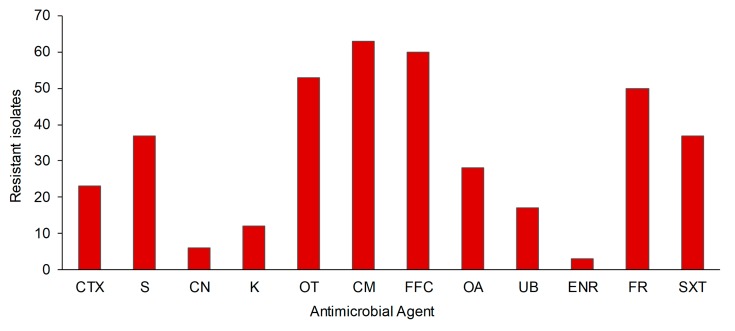
Antibacterial resistance of studied strain (*n* = 65) used in the study. CTX, Cefotaxime; S, Streptomycin; CN, Gentamicin; K, Kanamycin; OT, Oxytetracycline; CM, Chloramphenicol; FFC, Florfenicol; OA, Oxolinic acid; UB, Flumequine; ENR, Enrofloxacin; FR, Furazolidone; SXT, Sulfamethoxazole-trimethoprim.

A high incidence of resistance to the antibacterials chloramphenicol, florfenicol, and oxytetracycline (96.9%, 92.3%, and 81.5%, respectively) as well as a low incidence of resistance to gentamicin, furazolidone, and enrofloxacin (9.2%, 7.7%, and 4.6%, respectively) were detected among the isolates (Figure 1). In addition, a high occurrence of multiresistance or resistance to at least three classes of antibacterials was observed (63 isolates, 96%), showing a high proportion of isolates (55 isolates, 84%) presenting simultaneous resistance to five or more antibiotics. It was noted that a large number of isolates (48 isolates, 73%) exhibited simultaneous resistance to florfenicol and oxytetracycline and were mainly resistant to six or more antibiotics. Although no breakpoint values to categorize flumequine resistance are currently stated, all isolates categorized as resistant (17 isolates) using antibiogram results exhibited MIC values of ≥16 µg/mL (Table 2). A total of 28 isolates were resistant to oxolinic acid including 17 isolates resistant to flumequine and 10 isolates exhibiting a reduced susceptibility to flumequine (MIC of 8 µg/mL).

The MIC values of flumequine for the analyzed isolates ranged from 0.25 µg/mL to >64 µg/mL, with MIC_50_ and MIC_90_ values of 8 µg/mL and 64 µg/mL, respectively. The highest MIC values (≥64 µg/mL) were observed in six *Pseudomonas* isolates, one *Kluyvera* isolate, and one *Rahnella* isolate (Table 2). In addition, other high MIC values (32 µg/mL) were observed in two *Pseudomonas* isolates, whereas seven isolates of *Pseudomonas* exhibited MIC values of 16 µg/mL (Table 2). A number of 17 isolates were resistant to flumequine, exhibiting MIC values of ≥16 μg/mL and belonging to the *Pseudomonas* (15 isolates), *Rahnella* (one isolate), and *Kluyvera* (one isolate) genera, whereas 29 isolates exhibited a reduced susceptibility to flumequine (MIC of 4–8 µg/mL). Otherwise, 19 isolates exhibiting MIC values of ≤2 μg/mL and categorized as susceptible, but showing various levels of susceptibility (ranging from 0.25 to 2 µg/mL) were observed (Table 2).

### 3.3. Activity of Efflux Pumps

The results showed that the efflux pump inhibitor (EPI) decreased the MIC values of flumequine in a high percentage of the studied isolates (75%) (Table 2), demonstrating the active participation of efflux systems in quinolone resistance exhibited by isolates recovered from salmonid farms. In the presence of the EPI, the MIC values ranged from 0.0625 µg/mL to >64 µg/mL, with MIC_50_ and MIC_90_ values of 1 µg/mL and 8 µg/mL, respectively. Among the isolates exhibiting high MIC values (≥16 µg/mL), only the isolates Kluyvera intermedia OP29 and Rahnella aquatilis FM7 maintained MICs ≥64 µg/mL, and Pseudomonas vranovensis FR20 and Pseudomonas azotoformans FR27 (MIC of 16 µg/mL) maintained their MIC values in the presence of the EPI (Table 2). When the EPI was included in the MIC assay, the MIC values of 24 and 11 isolates were reduced by four and three times, respectively, whereas an MIC reduction of two times was detected in seven isolates. Otherwise, 16 isolates maintained their MIC values (Table 2). Among the 17 flumequine-resistant isolates, an increase in the susceptibility to flumequine was observed in 10 isolates, suggesting the occurrence of active efflux pumps, whereas the remaining seven isolates apparently did not exhibit efflux pump-mediated activity (Table 2). Furthermore, at least 43 isolates (66%) exhibiting reduced susceptibility or resistance to flumequine showed E_max_ values ≥4 (Table 2). Otherwise, as shown in Table 2, EPI induced hypersusceptibility to flumequine among an important number of fully susceptible isolates (15 out of 19). MIC values of flumequine of Escherichia coli ATCC 25922, were 0.5 µg/mL in the presence and absence of the EPI, within the acceptable range stated by CLSI [56].

### 3.4. Mutations in Quinolone Targets

Among the 17 flumequine-resistant isolates, no significant differences between the proportion of efflux systems and the presence of chromosomal mutations in the DNA gyrase (*p* = 0.6276) were observed. Thus, the probability of finding a resistance mechanism mediated by efflux systems or chromosomal mutations among the resistant isolates was not significantly different.

The detected amino acid substitutions exhibited by the flumequine-resistant isolates are shown in Table 3. A high number of flumequine-resistant isolates exhibited one to three mutations in the GyrB subunit of the DNA gyrase (14 isolates), and among these, five isolates showed a double mutation, leading to an amino acid substitution at positions 400 and 413 (according to the *Escherichia coli* numbering of protein sequence, which resulted in a Leu-to-Ile and Arg-to-Lys change, respectively), whereas the other two resistant isolates also exhibited a third mutation at position 423, resulting in a Val-to-Gly substitution. Otherwise, seven resistant isolates exhibited a single mutation at position 417, resulting in a Leu-to-His change (Table 3).

Only the isolates *Pseudomonas fluorescens* 275, *Kluyvera intermedia* OP29, and *Rahnella aquatilis* FM7 exhibited a mutation in the GyrA subunit, leading to a single amino acid substitution of serine to isoleucine at position 83, and showing the highest MIC values, despite the absence of efflux pump activity. Only four resistant isolates showed a mutation in the ParC protein subunit of topoisomerase IV, and of these, *P. fluorescens* 275 and *R. aquatilis* FM7 also harbored a double mutation in the GyrA and GyrB subunits, whereas *K. intermedia* OP29 harbored a single mutation in GyrA, and *Pseudomonas libanensis* FP37 showed three mutations in the GyrB subunit; thus, no resistant mutants with a mutation in ParC alone were observed, and the amino acid substitution Ser-80 to either Ile or Leu (*Escherichia coli* numbering) was observed in three of these resistant isolates (Table 3).

### 3.5. Genes Encoding for Quinolone Resistance

None of the 65 studied isolates carried any of the assayed *aac(6′)-Ib-cr*, *qepA*, *oqxA, qnrA, qnrS, qnrD,* and *qnrC* genes, which confer low-level resistance to fluoroquinolones, and only the strain *Citrobacter gillenii* FP75 was found to carry a new variant of the *qnrB* gene (*qnrB89*). The complete sequence of this gene exhibited a similarity with the *qnrB* gene of 82% and 90% at the nucleotide and amino acid sequence level, respectively (unpublished results).

## 4. Discussion

The projected worldwide use of antibiotics in livestock is approximately 106,000 tons for 2030 [57]. In the aquaculture industry, the use of antibiotics has been commonly adopted as the first choice strategy for the control of bacterial fish pathogens, for which no effective vaccines are currently available. The intracellular pathogen *P. salmonis*, which is currently the most important cause of losses in marine Chilean salmon farms [29], is consequently becoming the main target for antibacterial therapy in Chilean salmon farms [1].

The use of quinolones is currently of great importance for human health, but they are also commonly used in aquaculture [2,17,58]. Although the use of quinolones has decreased in the Chilean farming industry, the occurrence of resistant bacteria in water and sediments impacted by fish farms has been previously reported [19,20,21,59]. Chile is the world’s second largest salmon producer, and quinolones, mainly flumequine, with an annual use rate of 1% (3.75 tons) in marine farms for 2017, are still used in this industry [1]. Furthermore, it has been reported that quinolones used in fish culture have a high persistence in sediments, evidencing that flumequine and oxolinic acid can persist in the surface sedimentary layer for at least 60 and 151 days, respectively, whereas in deeper sedimentary layers, these antibacterials can persist for more than 300 days [60,61].

The analysis of susceptibility to different antimicrobials revealed that a high percentage of studied isolates (96%) showed antimicrobial multiresistance, as was previously reported for isolates recovered from Chilean salmon farms [19,20,40]. The results of the present study showed a high occurrence of resistance to phenicols and tetracyclines, with 96.9% and 92.3% of the isolates exhibiting resistance to chloramphenicol and florfenicol, respectively, and 81.5% of isolates resistant to oxytetracycline. These results suggest the occurrence of a selective process as a consequence of the use of antimicrobials in Chilean fish farms, considering that a high percentage of the isolates (73.8%) exhibited simultaneous resistance to florfenicol and oxytetracycline, which are currently the most used antibiotics in Chilean salmon farms [1,2].

As previously mentioned, the most commonly reported mechanisms of resistance to quinolones include mutations in the antibiotic target enzymes and a decrease in the antibiotic accumulation within the bacteria by the activity of efflux systems [15]. In this study, 42 isolates (64.6%) showed efflux system activity conferring at least a two times decrease in the MIC value of flumequine, showing that significant resistance as well as a decreased susceptibility to flumequine are frequently mediated by efflux mechanisms. A high proportion of isolates exhibiting resistance to flumequine (MIC ≥ 16 µg/mL) harbored mutations in their DNA gyrase genes mostly in the *gyrB* gene, and most of them evidenced an efflux pump activity.

DNA gyrase has been described as the main target of quinolone action in Gram-negative bacteria [46]; thus, the presence of mutations in this enzyme is a critical factor in the emergence of high-level resistance [46,62]. In this study, only a single mutation in the GyrA subunit of the DNA gyrase of three resistant isolates was observed, corresponding to an amino acid substitution of serine by isoleucine at position 83. It must be noted that the substitution of serine 83 of the GyrA protein subunit has been widely described as conferring a high-level resistance to quinolones among various species including clinical *E. coli* isolates [13,43] and the fish pathogen *Aeromonas salmonicida* [63]. All of these isolates also harbored an amino acid substitution of serine by isoleucine or leucine at position 80 of the ParC subunit of topoisomerase IV. The change of the amino acid serine at position 80 by isoleucine or leucine is a non-conserved amino acid change, producing a modification in the external structure of the amino acid skeleton of the protein, thus decreasing the enzymatic affinity for the antibiotic, as was previously described in clinical isolates of *Staphylococcus aureus* and *Escherichia coli* [64]. Furthermore, it has been reported that simultaneous mutations in GyrA and ParC can confer high-level resistance to fluoroquinolones [61], as was observed in the isolates *K. intermedia* OP29, *R. aquatilis* FM7 and *P. fluorescens* 275, which showed mutations in both enzymes and exhibited the highest MIC values for flumequine, which was not affected by the pump inhibitor. It must be noted that *R. aquatilis* has been previously reported as a causative agent of sepsis in immunocompromised and immunocompetent human patients [65,66].

Giraud et al. [41] found a high correlation between the presence of a mutation at codon 87 of *gyr*A leading to an Asp-87 » Asn substitution and the level of resistance to quinolones among *A. salmonicida* isolates, but in this study, no presence of a mutation at codon 87 of *gyr*A in the studied isolates were observed. In contrast to many previous studies [41,63], the occurrence of *gyr*A mutations in most of the resistant isolates was unusual, thus requiring further studies to explain this phenomenon.

When flumequine-resistant isolates exhibited a single (Leu-417 to His-417) or double (Leu-400 to Ileu-400 and Arg-413 to Lys-413) mutation in the GyrB subunit of DNA gyrase, MIC values decreased by at least three times when the efflux pump inhibitor was administered, suggesting that these mutations in GyrB could be causative of an increase in the level of resistance to fluoroquinolones as a consequence of a synergistic activity with efflux pumps among the Chilean salmonid farming-associated bacteria. Thus, novel mutations producing the amino acid changes observed in the GyrB subunit are probably not directly associated with high-level resistance as occurs with mutations in the GyrA and ParC subunits. Most probably, a high-level of resistance is only observed when active efflux and *gyr*B mutations are contributing independently to phenotypic flumequine resistance. Similar results have been observed in various bacterial species of clinical importance, where amino acid changes in GyrB do not confer fluoroquinolone resistance [46,64].

As previously mentioned, the efflux systems were active in 75% of the studied isolates, demonstrating their role as the main mechanisms of resistance to quinolones in the resistant microbiota associated with Chilean salmon farms. The results suggests that active efflux contributes significantly to the intrinsic resistance of a high number of isolates, in agreement with Lomovskaya et al. [67], who previously demonstrated that inhibition of efflux pumps significantly decreased the level of intrinsic resistance in *P. aeruginosa.* It has been previously noted that approximately 10% of the genes carried by a bacterium encode efflux systems [68]. Thus, efflux systems are an integral component of bacterial membranes, usually showing a high distribution among the different bacterial genera, so it is not surprising that they are active in both low and high levels of resistance to quinolones [69].

Frequently, the presence of efflux systems with enhanced expression as a consequence of an antibiotic selection pressure can be accompanied by chromosomal mutations producing a synergistic activity, consequently conferring a high-level of resistance, as was previously demonstrated [70] and exhibited by some isolates included in this study such as *P. fluorescens* 275, which exhibited an MIC value of >64 µg/mL, mediated both by chromosomal mutations in GyrA and by the activity of efflux systems because this value decreased to 32 µg/mL in the presence of the efflux pump inhibitor. However, isolates *P. vranovensis* FR20 and *P. azotoformans* FR27 neither showed amino acid changes in DNA gyrase and topoisomerase IV, nor a decrease in their flumequine MIC values in the presence of the efflux pump inhibitor, suggesting that resistance is mediated by a mechanism not currently described or by the activity of other efflux pumps not inhibited by the used pump inhibitor. Regarding the efflux pumps inhibitors, considering that in the study the Phe-Arg-βNA efflux pump inhibitor was the only one assayed, it was not possible to elucidate the effect of other efflux pump inhibitors on the flumequine susceptibility of isolates. Otherwise, it must be noted that a decrease in the MIC values after the addition of efflux pump inhibitors was not able to confirm the existence of efflux pumps inhibitors, thus it can be presumed that efflux pumps play a major role in the intrinsic resistance to flumequine among the bacterial isolates.

Considering that *qnr* genes encoding for quinolone target protection are unable by themselves to confer high-level resistance to quinolones and are only able to decrease quinolone susceptibility, their detection is more difficult to achieve because most studies only include bacterial isolates that are selected by their expression of resistance to quinolones. Consequently, it must be noted that most of these studies usually do not include isolates exhibiting low levels of resistance to quinolones; thus, their role and prevalence in environments impacted by fish farms are probably underestimated. In this trend, these studies could only detect *qnr* genes in isolates harboring *qnr* genes when they were combined with other mechanisms such as chromosomal resistance mutations. In this study, only the isolate *Citrobacter gilleni* FP75 was found to carry a variant of *qnrB* gene (*qnrB89*), most probably located in the chromosome, as has been extensively reported for other variants of the *qnrB* genes found in *Citrobacter* spp. [71,72,73].

Furthermore, it would be interesting to detect the occurrence of the *crpP* gene mainly among the *Pseudomonas* species, which encodes for a protein capable of specifically conferring resistance to ciprofloxacin through an ATP-dependent mechanism that involves phosphorylation of the antibiotic [74]. This novel protein has never been detected among bacteria associated with aquaculture, and its activity on flumequine remains unknown.

## 5. Conclusions

This study provides evidence for the importance of the Chilean salmon farming-related environment as a reservoir of bacteria exhibiting resistance as well as a reduction in the levels of susceptibility to quinolones, showing that bacterial resistance to quinolones in isolates associated with Chilean salmon farming is mainly mediated by nonspecific efflux pumps and to a much lesser extent by chromosomal mutations in the GyrA and ParC quinolone targets, whereas a high predominance of mutations in the subunit GyrB of DNA gyrase was observed and commonly associated with presumptive efflux activity. The carriage of plasmid-encoded efflux pump genes or transferable extrachromosomal genes encoding for quinolone target protection such as *qnr* genes, which provide low-level fluoroquinolone resistance, is apparently rare, In conclusion, microbiota associated with the Chilean salmon farm environment and exhibiting resistance or low susceptibility to quinolones, are mostly composed by the *Pseudomonas* species that are apparently mostly intrinsic, thus not contributing to the spread of horizontally transferred genes encoding for resistance to quinolones in these environments.

## Figures and Tables

**Table 1:** Primers used in the study.

Gene	Forward (5′-3′)	Reverse (5′–3′)	Amplicon Size (bp)	Reference
*16S*	AGAGTTTGATCCTGGCTCAG	GGTTACCTTGTTACGACTT	1200–1500	[32]
*gyrA* *	AAATCTGCCCGTGTCGTTGGT	GCCATACCTACGGCGATACC	344	[43]
	TACACCGGTCAACATTGAGG	TTAATGATTGCCGCCGTCGG	629	[43]
	GAGCTGGGCAACGACTGGAACAAGCCC	GATACCGCTGGAACCGTTGACCAGCAG	363	[44]
*gyrB* *	GGACAAAGAAGGCTACAGCA	CGTCGCGTTGTACTCAGATA	850	[45]
	TGCTGTGGTAGCGCAGTTTA	GCAGATGAACGAACTGCTGA	425	[46]
	GTGAAATGACGCGTCGTAAG	CGAATGTGTGAACCATCGAC	355	[46]
*parC* *	CTGAATGCCAGCGCCAAATT	GCGAACGATTTCGGATCGTC	168	[47]
	GTCACTTTTTGCARCTCYTC	TGAGCAGAAACTGCTGATG	384	This study
	GGCGCAGTTTGATCTTACG	ATAACGCCCGTGTGATGC	250	This study
	CAACTACTCGATGTACGTVAT	CGAAGGACTTGGGRTCRT	290	This study
*parE* *	GACCGAAAGCTACGTCAACC	GTTCGGATCAAGCGTGGTTT	932	[48]
	ATCTTCCGCAGACAGCTTCA	GGTAAACGCAATACCGGHAC	450	This study
	ATATCTTCCGCCGACAGCTT	GGACCAGCGTCCACTTCTG	440	This study
	GATCAGGTTGACGTARCTYT	GTCGGCAAGCGCAAYACC	330	This study
*qnrA*	ATTTCTCACGCCAGGATTTG	GATCGGCAAAGGTTAGGTCA	516	[49]
*qnrB*	GATCGTGAAAGCCAGAAAGG	ACGATGCCTGGTAGTTGTCC	469	[49]
*qnrC*	GGGTTGTACATTTATTGA	TCCACTTTACGAGGTTCT	447	[50]
*qnrD*	CGAGATCAATTTACGGGGAATA	AACAAGCTGAAGCGCCTG	582	[51]
*qnrS*	GCAAGTTCATTGAACAGGGT	TCTAAACCGTCGAGTTCGGCG	428	[52]
*qepA*	AACTGCTTGAGCCCGTAGAT	GTCTACGCCATGGACCTCAC	596	[53]
*oqxA*	CTCGGCGCGATGATGCT	CCACTCTTCACGGGAGACGA	390	[53]
*aac(6′)-lb-cr*	TTGCGATGCTCTATGAGTGGCTA	CTCGAATGCCTGGCGTGTTT	482	[53]

* Chromosomal mutations were investigated only in isolates exhibiting resistance to flumequine according to their antibiograms, and MIC values of ≥16 µg/mL.

**Table 2:** Identification, source, antibiotic resistance, and minimum inhibitory concentrations (MIC) of flumequine (FLU) of isolates.

Strain	Source	Access No	Closest Species (% Identity)	MIC FLU (µg/mL)		*E*_max_	Resistance Pattern
				Without EPI	With EPI		
275	Fingerling mucus	MH620734.1	*Pseudomonas fluorescens* (99.7)	>64	32	>2	CM-FFC-OT-OA-UB-ENR-FR-SXT
FB13	Fingerling mucus	KX279647.1	*Pseudomonas putida* (99.9)	>64	8	>8	CTX-CM-FFC-OA-UB-FR-SXT
FM7	Fingerling mucus	MH620756.1	*Rahnella aquatilis* (99.7)	>64	>64	ND	S-CM-FFC-OT-OA-UB-FR
FR34	Fingerling mucus	KX279664.1	*Pseudomonas baetica* (99.5)	>64	0.5	>128	CTX-CM-FFC-OA-UB-ENR-FR-SXT
OP29	Under-cage sediment	KX279666.1	*Kluyvera intermedia* (99.6)	>64	>64	ND	S-CM-FFC-OT-OA-UB
FB15	Fingerling mucus	KX279648.1	*Pseudomonas putida* (99.9)	64	4	16	CM-FFC-OA-UB-FR-SXT
FE24	Intestinal content	MH620733.1	*Pseudomonas baetica* (99.7)	64	0.5	128	CTX-CM-FFC-OA-UB-FR-SXT
FP37	Fingerling mucus	KX279659.1	*Pseudomonas libanensis* (99.9)	64	8	8	S-K-CM-FFC-OT-OA-UB-FR-SXT
FB90	Fingerling mucus	MH620722.1	*Pseudomonas oryzihabitans* (99.5)	32	4	8	CM-FFC-OT-OA-UB-FR-SXT
FP42	Fingerling mucus	MH620723.1	*Pseudomonas oryzihabitans* (99.3)	32	1	32	CM-FFC-OA-UB-FR-SXT
118	Fingerling mucus	MH620730.1	*Pseudomonas migulae* (99.2)	16	0.25	64	CM-FFC-OT-OA-UB-FR-SXT
FM4	Fingerling mucus	KX279655.1	*Pseudomonas putida* (99.6)	16	1	16	CTX- S-CM-FFC-OT-OA-UB-FR-SXT
FM15	Fingerling mucus	KX279657.1	*Pseudomonas putida* (99.2)	16	1	16	S-CN-CM-FFC-OT-OA-UB-ENR-FR-SXT
FM22	Cage water	KX279658.1	*Pseudomonas japonica* (99.9)	16	1	16	CTX-S-CN-K-CM-FFC-OT-OA-UB-FR-SXT
FP68	Fingerling mucus	MH620724.1	*Pseudomonas migulae* (99.5)	16	1	16	CTX-CM-FFC-OA-UB-FR-SXT
FR20	Fingerling mucus	MH620720.1	*Pseudomonas vranovensis* (100.0)	16	16	1	CTX-S-CM-FFC-OA-UB-FR-SXT
FR27	Fingerling mucus	KX279663.1	*Pseudomonas azotoformans* (100.0)	16	16	1	CTX-S-CM-FFC-OA-UB-FR-SXT
133	Fingerling mucus	MH620717.1	*Pseudomonas gessardii* (99.8)	8	8	1	CM-FFC-OT-OA-FR-SXT
144	Fingerling mucus	MH620718.1	*Pseudomonas gessardii* (99.7)	8	8	1	CTX-S-CM-FFC-OT-FR
145	Fingerling mucus	MH620729.1	*Pseudomonas fluorescens* (99.6)	8	8	1	CM-FFC-OT-FR-SXT
167	Fingerling mucus	MH620747.1	*Acinetobacter johnsonii* (99.0)	8	8	1	OT-OA
C2	Effluent	MH620749.1	*Stenotrophomonas maltophilia* (99.5)	8	4	2	S-CM-OT-OA
C6	Influent	MH620748.1	*Stenotrophomonas rhizophila* (99.1)	8	2	4	CTX-S-CM-FFC-OT-OA-FR
FE22	Intestinal content	MH620738.1	*Kluyvera intermedia* (99.4)	8	8	1	S-CM-FFC-OT-FR
FE23	Intestinal content	MH620739.1	*Kluyvera intermedia* (98.7)	8	0.125	64	S-K-CM-FFC-OT-FR
FF10	Fingerling mucus	MH620726.1	*Pseudomonas lurida* (99.6)	8	1	8	CTX-CM-FFC-OA-FR-SXT
FF32	Cage water	KX279652.1	*Pseudomonas putida* (99.6)	8	0.125	64	CM-FFC-OT-FR-SXT
FM2	Cage water	KX279653.1	*Sphingobacterium anhuiense* (99.3)	8	4	2	S-CN-K-CM-FFC-OT-OA-FR-SXT
FM26	Fingerling mucus	MH620736.1	*Kluyvera intermedia* (99.8)	8	4	2	S-CN-K-CM-FFC-OT-OA-FR
FR50	Fingerling mucus	MH620721.1	*Pseudomonas lini* (100.0)	8	8	1	CTX-CM-FFC-FR-SXT
OT30	Fingerling mucus	MH620725.1	*Pseudomonas poae* (100.0)	8	1	8	CTX-CM-FFC-OT-OA-FR-SXT
OT42	Fingerling mucus	KX279667.1	*Pseudomonas fluorescens* (99.9)	8	0.5	16	CTX-CM-FFC-OT-FR-SXT
SX37	Under-cage sediment	MH620727.1	*Pseudomonas putida* (99.6)	8	0.125	64	S-CM-FFC-OA-FR-SXT
SX53	Fingerling mucus	MH620731.1	*Pseudomonas reinekei* (99.9)	8	0.125	64	CM-FFC-OT-FR-SXT
Q20	Fingerling mucus	KX279669.1	*Pseudomonas fluorescens* (99.8)	8	0.5	16	CTX-S-CM-FFC-OT-OA-FR-SXT
Q23	Fingerling mucus	KX279670.1	*Pseudomonas fluorescens* (100.0)	8	0.5	16	CTX-S-CM-FFC-OT-FR-SXT
177	Fingerling mucus	MH620735.1	*Pseudomonas fluorescens* (99.1)	4	0.5	8	CM-FFC-OT-FR
227	Fingerling mucus	MH620728.1	*Pseudomonas fluorescens* (100.0)	4	0.125	32	CM-FFC-OT-FR-SXT
264	Effluent	MH620719.1	*Pseudomonas migulae* (99.6)	4	0.125	32	CTX-CM-FFC-OT-FR-SXT
CH3	Effluent	MH620740.1	*Kluyvera intermedia* (99.7)	4	0.25	16	S-CM-OT
FB133	Fingerling mucus	MH620751.1	*Lelliottia amnigena* (99.2)	4	0.5	8	S-CM-FFC-OT
FE12	Fingerling mucus	MH620745.1	*Hafnia alvei* (99.2)	4	4	1	CTX-S-CM-FFC-OT-FR
FE15	Fingerling mucus	MH620737.1	*Kluyvera intermedia* (99.2)	4	4	1	S-K-CM-FFC-OT
Q11	Rearing tank water	KX279668.1	*Pseudomonas migulae* (99.8)	4	0.0625	64	CTX-CM-FFC-OT-FR-SXT
Q73	Influent	MH620759.1	*Escherichia coli* (99.4)	4	0.0625	64	S-CM-FFC-OT-SXT
SX57	Fingerling mucus	MH620732.1	*Pseudomonas fluorescens* (99.6)	4	0.5	8	CTX-S-CM-FFC-OA-FR
CH83	Effluent	MH620755.1	*Serratia liquefaciens* (99.5)	2	1	2	CM-FFC-OT-FR
FB38	Fingerling mucus	MH620742.1	*Citrobacter freundii* (99.5)	2	2	1	S-K-CM-FFC-OT
FB98	Fingerling mucus	KX279649.1	*Citrobacter gillenii* (99.8)	2	2	1	S-K-CM-FFC-OT-SXT
Q61	Effluent	MH620758.1	*Providencia vermicola* (97.2)	2	0.25	8	CM-OT-FR
Q64	Effluent	KX279671.1	*Pseudomonas syringae* (99.6)	2	0.0625	32	CM-FFC-OT-FR-SXT
FB1	Fingerling mucus	MH620750.1	*Lelliottia amnigena* (97.1)	1	0.0625	16	S-CM-FFC-OT
FB11	Fingerling mucus	MH620741.1	*Citrobacter gillenii* (99.8)	1	0.25	4	S-K-CM-FFC-OT
FB96	Fingerling mucus	MH620752.1	*Lelliottia amnigena* (97.3)	1	0.0625	16	S-CM-FFC-OT
FE21	Intestinal content	MH620754.1	*Serratia myotis* (99.3)	1	1	1	S-K-CM-FFC-OT-FR
FM1	Cage water	MH620753.1	*Enterobacter ludwigii* (98.3)	1	1	1	S-CN-K-CM-FFC-OT-FR
OP21	Under-cage sediment	MH620743.1	*Citrobacter braakii* (99.5)	1	0.25	4	S-K-CM-FFC-OT-SXT
Q75	Pelletized feed	KX279673.1	*Acinetobacter johnsonii* (99.7)	1	0.25	4	CM-FFC-OT-FR
FE20	Intestinal content	MH620746.1	*Hafnia alvei* (99.7)	0.5	0.125	4	CTX-S-CM-FFC-OT-FR
FM3	Cage water	MH620757.1	*Comamonas jiangduensis* (99.9)	0.5	0.125	4	S-CM-FFC-OT-SXT
233	Cage water	MH424518.1	*Pseudomonas putida* (98.4)	0.25	0.0625	4	CTX-CM-FFC-OT-FR-SXT
C11	Pelletized feed	MH620761.1	*Morganella psychrotolerans* (99.6)	0.25	0.125	2	OT
FE11	Under-cage sediment	MH620744.1	*Hafnia alvei* (99.6)	0.25	0.0625	4	CTX-S-CM-FFC-OT-FR
FM16	Fingerling mucus	MH620760.1	*Leclercia adecarboxylata* (98.2)	0.25	0.0625	4	S-CN-CM-FFC-OT-FR
FP75	Under-cage sediment	KX279662.1	*Citrobacter gillenii* (99.6)	0.25	0.125	2	S-CM-FFC-OT-FR-SXT

EPI: Efflux pump inhibitor; *E*_max_: MIC without EPI/MIC in the presence of EPI; ND: Not determined; CTX: Cefotaxime; S: Streptomycin; K: Kanamycin; CN: Gentamicin; CM: Chloramphenicol; FFC: Florfenicol; OT: Oxytetracycline; OA: Oxolinic acid; UB: Flumequine; ENR: Enrofloxacin; FR: Furazolidone; SXT: Sulfamethoxazole-trimethoprim.

**Table 3:** Aminoacidic substitutions in isolates exhibiting resistance to flumequine.

Strain	Aminoacidic Substitution ^a^	
	DNA Gyrase	Topoisomerase IV
*Pseudomonas fluorescens* 275	GyrA: S83 by I; GyrB: L417 by H	ParC: Y74 by F; S80 by L; P98 by T
*Pseudomonas putida* FB13	GyrB: L400 by I; R413 by K; V423 by G	None
*Rahnella aquatilis* FM7	GyrA: S83 by I; GyrB: L417 by H	ParC: S80 by I
*Pseudomonas baetica* FR34	GyrB: L417 by H	None
*Kluyvera intermedia* OP29	GyrA: S83 by I	ParC: S80 by I
*Pseudomonas putida* FB15	GyrB: L417 by H	None
*Pseudomonas baetica* FE24	GyrB: L417 by H	None
*Pseudomonas libanensis* FP37	GyrB: L400 by I; R413 by K; V423 by G	ParC: D101 by N
*Pseudomonas oryzihabitans* FB90	GyrB: L417 by H	None
*Pseudomonas* sp. FP42	GyrB: L400 by I; R413 by K	None
*Pseudomonas* sp. 118	GyrB: L417 by H	None
*Pseudomonas putida* FM4	GyrB: L400 by I; R413 by K	None
*Pseudomonas* sp. FM15	GyrB: L400 by I; R413 by K	None
*Pseudomonas japonica* FM22	GyrB: L400 by I; R413 by K	None
*Pseudomonas migulae* FP68	GyrB: L400 by I; R413 by K	None
*Pseudomonas vranovensis* FR20	None	None
*Pseudomonas azotoformans* FR27	None	None

^a^: S, Serine; I, Isoleucine; L, Leucine; H, Histidine, Y, Tyrosine; F, Phenyl-alanine; P, Proline; T, Threonine; R, Arginine; K, Lysine; V, Valine; G, Glycine; D, Aspartic acid; N, Asparagine.

## References

[1] SERNAPESCA Informe Sobre Uso de Antimicrobianos en la Salmonicultura Nacional 2017. http://www.sernapesca.cl/sites/default/files/informe_sobre_uso_de_antimicrobianos_2017.pdf.

[2] Miranda C.D., Godoy F.A., Lee M. (2018). Current status of the use of antibiotics and the antimicrobial resistance in the antimicrobial resistance in the *Chilean salmon* farms. Front. Microbiol..

[3] Miranda C.D., Monforts H.M.M., Keen P.L. (2012). Antimicrobial resistance in salmonid farming. Antimicrobial Resistance in the Environment.

[4] Henríquez P., Bohle H., Bustamante F., Bustos P., Mancilla M. (2015). Polymorphism in *gyrA* is associated to quinolones resistance in Chilean *Piscirickettsia salmonis* field isolates. J. Fish Dis..

[5] Henríquez P., Kaiser M., Bohle H., Bustos P., Mancilla M. (2016). Comprehensive antibiotic susceptibility profiling of Chilean *Piscirickettsia salmonis* field isolates. J. Fish Dis..

[6] Miranda C.D., Smith P., Rojas R., Contreras-Lynch S., Vega J.M.A. (2016). Antimicrobial susceptibility of *Flavobacterium psychrophilum* from Chilean salmon farms and their epidemiological cut-off values using agar dilution and disk diffusion methods. Front. Microbiol..

[7] Saavedra J., Grandón M., Villelobos-Gonzáles J., Bohle H., Bustos P., Mancilla M. (2018). Isolation, functional characterization and transmissibility of p3PS10, a multidrug resistance plasmid of the fish pathogen *Piscirickettsia salmonis*. Front. Microbiol..

[8] Dalhoff A. (2012). Global fluoroquinolone resistance epidemiology and implications for clinical use. Interdiscip. Perspect. Infect. Dis..

[9] Zheng F., Meng X.-Z. (2017). Research on pathogenic bacteria and antibiotic resistance of Enterobacteriaceae in hospitalized elderly patients. Biomed. Res..

[10] Baker S., Thomson N., Weil F.-X., Holt K.E. (2018). Genomic insights into the emergence and spread of antimicrobial-resistant bacterial pathogens. Science.

[11] Ashley R.E., Dittmore A., McPherson S.A., Turnbough C.L., Neuman K.C., Osheroff N. (2017). Activities of gyrase and topoisomerase IV on positively supercoiled DNA. Nucleic Acids Res..

[12] Correia S., Poeta P., Hébraud M., Capelo J.L., Igrejas G. (2017). Mechanisms of quinolone action and resistance: Where do we stand?. J. Med. Microbiol..

[13] Ruiz J. (2003). Mechanisms of resistance to quinolones: Target alterations, decreased accumulation and DNA gyrase protection. J. Antimicrob. Chemother..

[14] Aldred K.J., Kerns R.J., Osheroff N. (2014). Mechanism of quinolone action and resistance. Biochemistry.

[15] Fàbrega A., Madurga S., Giralt E., Vila J. (2009). Mechanism of action of and resistance to quinolones. Microb. Biotechnol..

[16] Hooper D.C., Jacoby G.A. (2015). Mechanisms of drug resistance: Quinolone resistance. Ann. N. Y. Acad. Sci..

[17] Sapkota A., Sapkota A.R., Kucharski M., Burke J., McKenzie S., Walker P., Lawrence R. (2008). Aquaculture practices and potential human health risks: Current knowledge and future priorities. Environ. Int..

[18] Cabello F.C., Godfrey H.P., Tomova A., Ivanova L., Dölz H., Millanao A., Buschmann A.H. (2013). Antimicrobial use in aquaculture re-examined: Its relevance to antimicrobial resistance and to animal and human health. Environ. Microbiol..

[19] Miranda C.D., Zemelman R. (2002). Antimicrobial multiresistance in bacteria isolated from freshwater Chilean salmon farms. Sci. Total Environ..

[20] Miranda C.D., Rojas R. (2007). Occurrence of florfenicol resistance in bacteria associated with two *Chilean salmon* farms with different history of antibacterial usage. Aquaculture.

[21] Buschmann A.H., Tomova A., López A., Maldonado M.A., Henríquez L.A., Ivanova L., Moy F., Godfrey H.P., Cabello F.C. (2012). Salmon aquaculture and antimicrobial resistance in the marine environment. PLoS ONE.

[22] Ishida Y., Ahmed A., Mahfouz N., Kimura T., El-Khodery S., Moawad A., Shimamoto T. (2010). Molecular analysis of antimicrobial resistance in Gram-negative bacteria isolated from fish farms in Egypt. J. Vet. Med. Sci..

[23] Takasu H., Suzuki S., Reungsang A., Pham H.V. (2011). Fluoroquinolone (FQ) contamination does not correlate with occurrence of FQ-resistant bacteria in aquatic environments of Vietnam and Thailand. Microbes Environ..

[24] Jiang H., Tang D., Liu Y., Zhang X., Zeng Z., Xu L., Hawkey P.M. (2012). Prevalence and characteristics of β-lactamase and plasmid-mediated quinolone resistance genes in *Escherichia coli* isolated from farmed fish in China. J. Antimicrob. Chemother..

[25] Tomova A., Ivanova L., Buschmann A.H., Godfrey H.P., Cabello F.C. (2018). Plasmid-mediated quinolone resistance (PMQR) genes and class 1 integrons in quinolone-resistant marine bacteria and clinical isolates of *Escherichia coli* from an aquacultural area. Microb. Ecol..

[26] Poirel L., Liard A., Rodriguez-Martinez J.M., Nordmann P. (2005). Vibrionaceae as a possible source of Qnr-like quinolone resistance determinants. J. Antimicrob. Chemother..

[27] Xiong X., Bromley E.H.C., Oelschlaeger P., Woolfson D.N., Spencer J. (2011). Structural insights into quinolone antibiotic resistance mediated by pentapeptide repeat proteins: Conserved surface loops direct the activity of a Qnr protein from a Gram-negative bacterium. Nucleic Acids Res..

[28] Pons M.J., Gomes C., Ruiz J. (2013). QnrVC, a new transferable Qnr-like family. Enferm. Infecc. Microbiol. Clin..

[29] SERNAPESCA Informe Sanitario de Salmonicultura en Centros Marinos 2016. http://www.sernapesca.cl/sites/default/files/informe_sanitario_salmonicultura_en_centros_marinos_2018_final.pdf.

[30] SERNAPESCA Informe Sobre Uso de Antimicrobianos en la Salmonicultura Nacional 2010. http://www.sernapesca.cl/sites/default/files/informe_sobre_uso_de_antimicrobianos_2010.pdf.

[31] Domínguez M., Miranda C.D., Fuentes O., de la Fuente M., Godoy F.A., Bello-Toledo H., González-Rocha G. (2019). Occurrence of transferable integrons and *sul* and *dfr* genes among sulfonamide-and/or trimethoprim-resistant bacteria isolated from Chilean salmonid farms. Front. Microbiol..

[32] Opazo R., Ortúzar F., Navarrete P., Espejo R., Romero J. (2012). Reduction of soybean meal non-starch polysaccharides and α-Galactosides by solid-state fermentation using cellulolytic bacteria obtained from different environments. PLoS ONE.

[33] Ribosomal Database Project. http://rdp.cme.msu.edu/.

[34] CLSI (2015). Methods for Dilution Antimicrobial Susceptibility Tests for Bacteria that Grow Aerobically; Approved Standard—Tenth Edition.

[35] CLSI (2006). Methods for Broth Dilution Susceptibility Testing of Bacteria Isolated from Aquatic Animals; Approved Guideline M49-A.

[36] European Committee on Antimicrobial Susceptibility Testing (EUCAST). https://mic.eucast.org/Eucast2/.

[37] CLSI (2015). Performance Standards for Antimicrobial Disk Susceptibility Test; Approved Standard—Twelfth Edition.

[38] CLSI (2006). Methods for Antimicrobial Disk Susceptibility Testing of Bacteria Isolated from Aquatic Animals; Approved Guideline VET03-A.

[39] CLSI (2018). Performance Standards for Antimicrobial Disk and Dilution Susceptibility Tests for Bacteria Isolated from Animals.

[40] Fernández-Alarcón C., Miranda C.D., Singer R.S., López Y., Rojas R., Bello H., Domínguez M., González-Rocha G. (2010). Detection of the *floR* gene in a diversity of florfenicol resistant Gram-negative bacilli from freshwater salmon farms in Chile. Zoonoses Public Health.

[41] Giraud E., Blanc G., Bouju-Albert A., Weill F.-X., Donnay-Moreno C. (2004). Mechanisms of quinolone resistance and clonal relationship among *Aeromonas salmonicida* strains isolated from reared fish with furunculosis. J. Med. Microbiol..

[42] Hall T.A. (1999). BioEdit: A user-friendly biological sequence alignment editor and analysis program for Windows 95/98/NT. Nucleic Acids Symp. Ser..

[43] Weigel L.M., Steward C.D., Tenover F.D. (1998). *gyrA* mutations associated with fluoroquinolone resistance in eight species of *Enterobacteriaceae*. Antimicrob. Agents Chemother..

[44] Brown J.C., Ames S.G. (1998). Quinolone resistance. Methods Mol. Med..

[45] Akasaka T., Tanaka M., Yamaguchi A., Sato K. (2001). Type II topoisomerase mutations in fluoroquinolone-resistant clinical strains of *Pseudomonas aeruginosa* isolated in 1998 and 1999: Role of target enzyme in mechanism of fluoroquinolone resistance. Antimicrob. Agents Chemother..

[46] De la Fuente M., Dauros P., Bello H., Domínguez M., Mella S., Sepúlveda M., Zemelman R., González G. (2007). Mutaciones en genes *gyrA* y *gyrB* en cepas de bacilos Gram negativos aislados en hospitales chilenos y su relación con la resistencia a fluoroquinolonas. Rev. Méd. Chile.

[47] Rodríguez-Martínez J.M., Velasco C., Pascual A., García I., Martínez-Martínez L. (2006). Correlation of quinolone resistance levels and differences in basal and quinolone-induced expression from three *qnrA*-containing plasmids. Clin. Microbiol. Infect..

[48] Komp P., Karlsson Å., Hughes D. (2003). Mutation rate and evolution of fluoroquinolone resistance in *Escherichia coli* isolates from patients with urinary tract infections. Antimicrob. Agents Chemother..

[49] Robicsek A., Strahilevitz J., Jacoby G.A., Macielag M., Abbanat D., Park C.H., Bush K., Hooper D.C. (2006). Fluoroquinolone modifying enzyme: A new adaptation of a common aminoglycoside acetyltransferase. Nat. Med..

[50] Wang M., Guo Q., Xu X., Wang X., Ye X., Wu S., Hooper D.C. (2009). New plasmid-mediated quinolone resistance gene, *qnrC*, found in a clinical isolate of *Proteus mirabilis*. Antimicrob. Agents Chemother..

[51] Cavaco L.M., Hasman H., Xia S., Aarestrup F.M. (2009). *qnrD*, a novel gene conferring transferable quinolone resistance in *Salmonella enterica* serovar Kentucky and Bovis morbificans strains of human origin. Antimicrob. Agents Chemother..

[52] Cattoir V., Poirel L., Mazel D., Soussy C.J., Nordmann P. (2007). *Vibrio splendidus* as the source of plasmid-mediated QnrS-like quinolone resistance determinants. Antimicrob. Agents Chemother..

[53] El-Badawy M.F., Tawakol W.M., El-Far S.W., Maghrabi I.A., Al-Ghamdi S.A., Mansy M.S., Ashour M.S., Shohayeb M.M. (2017). Molecular identification of aminoglycoside-modifying enzymes and plasmid-mediated quinolone resistance genes among *Klebsiella pneumoniae* clinical isolates recovered from Egyptian patients. Int. J. Microbiol..

[54] Albert M., Yagüe G., Fernández M., Viñuela L., Segovia M., Muñoz J.L. (2014). Prevalence of plasmid-mediated quinolone resistance determinants in extended-spectrum β-lactamase-producing and -non-producing enterobacteria in Spain. Int. J. Antimicrob. Agents.

[55] R Core Team (2016). R: A Language and Environment for Statistical Computing.

[56] CLSI (2014). Performance Standards for Antimicrobial Susceptibility Testing of Bacteria Isolated from Aquatic Animals.

[57] Van Boeckel T.P., Brower C., Gilbert M., Grenfell B.T., Levin S.A., Robinson T.P., Teillant A., Laxminarayan R. (2015). Global trends in antimicrobial use in food animals. Proc. Natl. Acad. Sci. USA.

[58] Lalumera G.M., Calamari D., Galli P., Castiglioni S., Crosa G., Fanelli R. (2004). Preliminary investigation on the environmental occurrence and effects of antibiotics used in aquaculture in Italy. Chemosphere.

[59] Shah S., Cabello F.C., L’Abée-Lund T., Tomova A., Godfrey H., Buschmann A., Sørum H. (2014). Antimicrobial resistance and antimicrobial resistance genes in marine bacteria from salmon aquaculture and non-aquaculture sites. Environ. Microbiol..

[60] Björklund H., Bondestam J., Bylund G. (1990). Residues of oxytetracycline in wild fish and sediments from fish farms. Aquaculture.

[61] Hektoen H., Berge J.A., Hormazabal V., Yndestad M. (1995). Persistence of antibacterial agents in marine sediments. Aquaculture.

[62] Jacoby G.A. (2005). Mechanisms of resistance to quinolones. Clin. Infect. Dis..

[63] Oppegaard H., Sørum H. (1994). *gyrA* mutation in quinolone-resistant isolates of the fish pathogen *Aeromonas salmonicida*. Antimicrob. Agents Chemother..

[64] Sierra J.M., Cabeza J.G., Ruiz M., Montero T., Hernandez J., Mensa J., Llagostera M., Vila J. (2005). The selection of resistance to and the mutagenicity of different fluoroquinolones in *Staphylococcus aureus* and *Streptococcus pneumoniae*. Clin. Microbiol. Infect..

[65] Chang C.L., Jeong J., Shin J.H., Lee E.Y., Son H.C. (1999). *Rahnella aquatilis* sepsis in an immunocompetent adult. J. Clin. Microbiol..

[66] Tash K. (2005). *Rahnella aquatilis* bacteremia from a suspected urinary source. J. Clin. Microbiol..

[67] Lomovskaya O., Warren M.S., Lee A., Galazzo J., Fronko R., Lee M., Blais J., Cho D., Chamberland S., Renau T. (2001). Identification and characterization of inhibitors of multidrug resistance efflux pumps in *Pseudomonas aeruginosa*: Novel agents for combination therapy. Antimicrob. Agents Chemother..

[68] Webber M.A., Piddock L.J. (2003). The importance of efflux pumps in bacterial antibiotic resistance. J. Antimicrob. Chemother..

[69] Tavio M., Vile J., Ruiz J., Martín-Sánchez A.M., Jiménez de Anta M.T. (2000). Decreased permeability and enhanced proton dependent active efflux in the development of resistance to quinolones in *Morganella morganii* strains. Int. J. Antimicrob. Agents.

[70] Taneja N., Mishra A., Kumar A., Verma G., Sharma M. (2015). Enhanced resistance to fluoroquinolones in laboratory-grown mutants and clinical isolates of *Shigella* due to synergism between efflux pump expression and mutations in quinolone resistance determining region. Indian J. Med. Res..

[71] Jacoby G.A., Griffin C.M., Hooper D.C. (2011). *Citrobacter* spp. as a source of *qnrB* alleles. Antimicrob. Agents Chemother..

[72] Saga T., Sabtcheva S., Mitsutake K., Ishii Y., Tateda K., Yamaguchi K., Kaku M. (2013). Characterization of *qnrB*-like genes in *Citrobacter* species of the American type culture collection. Antimicrob. Agents Chemother..

[73] Campos M.J., Palomo G., Hormeño L., Rodrigues A.P., Sánchez-Benito R., Píriz S., Quesada A. (2015). Detection of QnrB54 and its novel genetic context in *Citrobacter freundii* isolated from a clinical case. Antimicrob. Agents Chemother..

[74] Chávez-Jacobo V.M., Hernández-Ramírez K.C., Romo-Rodríguez P., Pérez-Gallardo R.V., Campos-García J., Gutiérrez-Corona J.F., García-Merinos J.P., Meza-Carmen V., Silva-Sánchez J., Ramírez-Díaz M.I. (2018). CrpP is a novel ciprofloxacin-modifying enzyme encoded by the *Pseudomonas aeruginosa* pUM505 plasmid. Antimicrob. Agents Chemother..

